# Phase II trial of S-1 and cisplatin with concurrent radiotherapy for locally advanced non-small-cell lung cancer

**DOI:** 10.1038/sj.bjc.6605152

**Published:** 2009-07-14

**Authors:** F Ohyanagi, N Yamamoto, A Horiike, H Harada, T Kozuka, H Murakami, K Gomi, T Takahashi, M Morota, T Nishimura, M Endo, Y Nakamura, A Tsuya, T Horai, M Nishio

**Affiliations:** 1 Thoracic Oncology Center, Cancer Institute Hospital, Japanese Foundation for Cancer Research, 3-10-6 Ariake, Koto-ku, Tokyo 135-8550, Japan; 2 Department of Thoracic Oncology, Shizuoka Cancer Center Hospital, 1007 Shimonagakubo, Nagaizumi-cho, Sunto-gun, Shizuoka 411-8777, Japan; 3 Division of Radiation Oncology, Shizuoka Cancer Center Hospital, Shizuoka, Japan 1007 Shimonagakubo, Nagaizumi-cho, Sunto-gun, Shizuoka, 411-8777, Japan; 4 Department of Radiation Oncology, Cancer Institute Hospital, Japanese Foundation for Cancer Research, 3-10-6 Ariake, Koto-ku, Tokyo 135-8550, Japan

**Keywords:** S-1, non-small-cell lung cancer, chemotherapy, radiotherapy, cisplatin

## Abstract

**Background:**

To assess the efficacy and safety of S-1 and cisplatin with concurrent thoracic radiation for unresectable stage III non-small-cell lung cancer (NSCLC).

**Methods:**

Eligible patients were 20–74 years old and had histologically or cytologically confirmed NSCLC, a performance status of 0–1, and no prior chemotherapy. Patients were treated with cisplatin (60 mg m^−2^ on day 1) and S-1 (orally at 40 mg m^−2^ per dose, b.i.d., on days 1–14), with the treatment repeated every 4 weeks for four cycles. Beginning on day 2, a 60-Gy thoracic radiation dose was delivered in 30 fractions.

**Results:**

Of 50 patients, 48 were eligible. Partial response was observed in 42 patients (87.5%; 95% CI: 79.1–96.9%). This regimen was well tolerated. Common toxicities included grade 3/4 neutropenia (32%), grade 3/4 leukopenia (32%), grade 3/4 thrombocytopenia (4%), grade 3 febrile neutropenia (6%), grade 3 oesophagitis (10%), and grade 3 pneumonitis (5%). Median progression-free survival was 12.0 months and median overall survival was 33.1 months. The 1- and 2-year survival rates were 89.5 and 56%, respectively.

**Conclusion:**

This chemotherapy regimen with concomitant radiotherapy is a promising treatment for locally advanced NSCLC because of its high response rates, good survival rates, and mild toxicities.

Lung cancer remains the leading cause of cancer-related deaths worldwide (Jemal *et al*, 2007). Approximately 30% of non-small-cell lung cancer (NSCLC) patients present with locally advanced lung cancer (van Meerbeeck, 2001). A number of randomised clinical trials support the conclusion that combined-modality approaches improve survival compared with radiotherapy alone for locally advanced lung cancer (Le Chevalier *et al*, 1991; Non-Small Cell Lung Cancer Collaborative Group, 1995). Depending on the strategy used, chemotherapy may have a cytotoxic role by eradicating distant micrometastases, may have a radiosensitising role by improving local control, or it perhaps may have a role in both effects. Recently, two randomised trials using cisplatin plus older agents from the 1980s compared sequential with concurrent chemoradiotherapy and showed superior survival with the concurrent approach (Furuse *et al*, 1999; Curran *et al*, 2003). However, distant metastases remain the major site of failure and it is also likely that more effective chemotherapy will be required for further improvement in results.

During the last decade, several new agents, such as paclitaxel, gemcitabine, vinorelbine, and docetaxel, have been proven to be more effective in metastatic NSCLC than the old regimens (Bunn and Kelly, 1998; Johnson, 1999; Hoffman *et al*, 2000).

Because of dose-limiting toxicities (Choy *et al*, 1998; Vokes *et al*, 2002; Gandara *et al*, 2003; Zatloukal *et al*, 2004; Fournel *et al*, 2005; Bedano *et al*, 2006; Kiura *et al*, 2008), these new agents have been tested using a reduced dose or split dose within the newer concurrent chemoradiation regimens. As yet, however, these new strategies have not been proven to be more effective than the older regimens.

S-1 is a new oral fluoropyrimidine agent designed to enhance anticancer activity and to reduce gastrointestinal toxicity through the combined use of an oral fluoropyrimidine agent (tegafur), a dihydropyrimidine dehydrogenase inhibitor (5-chloro-2,4-dihydroxypyridine), and an orotate phosphoribosyl transferase inhibitor (potassium oxonate; Shirasaka *et al*, 2000). S-1 has been shown to have one of the highest levels of response as a single agent for metastatic NSCLC (Kawahara *et al*, 2001). Moreover, two phase II trials of S-1 plus cisplatin for advanced NSCLC (stage IIIB without any indications for radiotherapy or stage IV) showed a response rate of 32.7–47% and a median survival time of 11–16 months. In addition, it shows very few severe gastrointestinal or haematological toxicities (Ichinose *et al*, 2004; Endo *et al*, 2006).

If the same doses of S-1 plus cisplatin used for advanced NSCLC could be concurrently used with thoracic radiotherapy (TRT), this regimen would be expected to have several advantages over previous regimens. First, the radiation-sensitising effect of 5-FU is well known clinically and S-1 also has been shown to act as a radiosensitiser in preclinical models (Fukushima *et al*, 1998; Harada *et al*, 2004). Second, in order to achieve radiosensitisation, prolonged exposure is desirable, and as such, oral administration is the most appealing route of administration. In addition, phase I studies have shown that the half-life of 5-FU after oral S-1 administration has been found to be markedly prolonged compared with that of 5-FU after intravenous administration (van Groeningen *et al*, 2000; Yamada *et al*, 2003). Therefore, we conducted a phase II trial using S-1 and cisplatin chemotherapy with concurrent TRT for locally advanced NSCLC.

## Patients and methods

### Patient selection

Patient eligibility requirements for enrollment in this study included cytologically or histologically documented NSCLC and measurable disease at a locally advanced stage IIIA or IIIB. Patients with T1–T3 and N2 disease if medically inoperable, T4 with any node size and extent, and those with N3 disease with any tumour involvement were eligible. Additional eligibility criteria included patient's age from 20 to 74 years, no prior treatment, Eastern Cooperative Oncology Group (ECOG) performance status of 0 or 1, projected life expectancy of at least 3 months, a leukocyte count of 4000–12 000 *μ*l^−1^, platelet count of ⩾100 000 *μ*l^−1^, haemoglobin level of ⩾9 g per 100 ml, serum bilirubin level of ⩽1.5 mg per 100 ml, serum aspartate aminotransferase and alanine aminotransferase levels of ⩽100 IU l^−1^, alkaline phosphatase level of twice the upper limit of normal or less, normal creatinine level, and partial pressure of arterial oxygen ⩾65 torr in room air.

For staging, all patients underwent a computed tomography (CT) scan of the thorax, including the upper abdomen, and either a brain CT scan or brain magnetic resonance imaging. A radioisotopic bone scan or a positron emission tomography (PET) scan was also performed in all of the patients. Mediastinoscopies were not performed and lymph node metastases were clinically diagnosed based on the results of a CT scan and/or PET scan.

Patients with significant pleural effusions or pericardial effusion seen on a CT scan were not eligible. Patients who were pregnant or who had concomitant serious diseases, a concomitant malignancy, or symptomatic cerebral involvement were excluded from the study. The institutional ethics committee of each of the participating institutions approved the protocol and all patients provided written informed consent before starting the study.

### Treatment plan

Before therapy was begun, a CT scan of the tumour in the chest was performed in order to determine tumour volume. Treatment in eligible patients began with the administration of two cycles of concurrent chemoradiotherapy that consisted of an oral administration of S-1 (40 mg m^−2^) twice daily for days 1–14 along with a 60-min intravenous infusion of cisplatin (60 mg m^−2^) on day 1 and then at 4-week intervals. For the radiation treatment, a 60-Gy dose was given in 30 fractions over a 6-week period. At 2 weeks after the last radiation treatment, patients received two more cycles of consolidation chemotherapy that consisted of the same doses and schedule as for the concurrent chemoradiotherapy. The oral doses of S-1 for each patient were assigned based on the body surface area. The three doses administered were 40 mg (body surface area <1.25 m^2^), 50 mg (1.25< body surface area <1.50 m^2^), and 60 mg (body surface area ⩾1.50 m^2^). Supportive care, which included adequate hydration and antiemetics, was provided. The use of granulocyte colony-stimulating factor during radiotherapy was not permitted.

If changes in the laboratory variables after the start of treatment occurred so that a leukocyte count of >3000 *μ*l^−1^ or a neutrophil count of >1500 *μ*l^−1^or any of the other entry eligibility criteria for the study were not met, subsequent courses of treatment were withheld until the noted abnormality had resolved. If there was no resolution of the abnormality within 6 weeks, the patient stopped the protocol treatment but was included in the study analysis.

The doses of S-1 were reduced in the event of any of the following toxicities during the previous treatment cycle: grade 4 haematological toxicity, or grade 3 or more non-haematological toxicity. For the subsequent courses, S-1 was reduced from 60, 50, or 40 mg twice daily to 50, 40, and 25 mg twice daily, respectively.

### Radiation therapy

Chest irradiation began concurrently on day 2 in all patients. Two different radiation target volumes were planned: the initial large-field target volume, which consisted of primary tumour and mediastinal lymph nodes, and the boost target volume, which consisted of the primary and involved nodes. Radiation therapy was delivered to the large-field volume (40 Gy) followed by a boost volume (20 Gy) with a dose of 2 Gy daily for 5 days each week for 6 weeks by a linear accelerator generating at least 4 MeV photons. The target volume of the primary tumour included the complete extent of the radiographically defined visible primary tumour and lymph nodes with a minimum 1.0- to 2.0-cm margin around the mass. Contralateral hilar lymph nodes were not included. The objectives were to restrict the relative volume of the normal lung treated with a dose of >20 Gy (V20) to <30%, and the maximum spinal cord dose was restricted to <50 Gy.

### Evaluation of response and toxicity

All eligible patients who received any portion of the treatment were considered assessable for response and toxicity. Chest X-rays, complete blood counts, and blood chemistry measurements were done weekly. The response was assessed based on the CT scan findings that initially had been used to define the extent of the tumour. The response was evaluated in accordance with the Response Evaluation Criteria in Solid Tumors (Therasse *et al*, 2000). The response was confirmed for at least 4 weeks (for a complete or partial response) or 6 weeks (for stable disease) after it was first documented.

Progression-free survival (PFS) was defined as the time from registration until objective tumour progression or death. Patients whose disease had not progressed at the time of study treatment discontinuation continued to be assessed until progression was documented. Overall survival (OS) was defined as the time from registration to death from any cause. Progression-free survival and OS were estimated by the Kaplan–Meier method. Adverse events were graded according to the National Cancer Institute Common Terminology Criteria for Adverse Events, Version 3.0.

Follow-up studies included a post-treatment CT scan at 4–6 weeks from the completion of all chemotherapy. Subsequently, follow-up was every 3 months for 3 years, then every 6 months until disease progression.

### Statistical analysis

The primary end point of the study was the response rate, which determined the sample size. For the first stage, Simon's minimax two-stage phase II design (Simon, 1989) was used to allow early termination if the preliminary results indicated minimal efficacy. We chose a 90% response rate as the experimental target level and a 70% response rate as the minimal target level with *α*- and *β*-errors of 0.1, resulting in a target of 25 patients. If 19 or more responses were observed among the 25 total assessable patients, the treatment was considered worthy of further consideration. For the second stage, an additional 25 patients were enrolled in order to achieve an overall maximum response rate of 90% with a confidence interval (CI) of 15%

## Results

### Patient characteristics

A total of 50 patients (43 men and 7 women) were enrolled in the trial between August 2005 and April 2007.

A radioisotopic bone scan was performed in 12 patients, a PET scan in 28 patients, and both were performed in 10 patients. In one patient, the PET scan did not show a bone metastasis, but a radioisotopic bone scan showed a vertebral bone metastasis before treatment. In another patient, a PET scan did not show an adrenal metastasis, but adrenal swelling was detected by an abdominal CT scan before treatment. Thus, the two patients were not eligible for the study.

Patient characteristics are summarised in Table 1. The median age was 63 years. The ECOG performance status was 0 for 72% and 1 for 28% of patients. Histological examinations indicated the presence of the following cancer cell types: adenocarcinoma, 60%; squamous, 28%; and other types, 12%. In total, 22 (44%) patients had stage IIIA disease; 26 (52%) patients had stage IIIB; and 2 (4%) patients had stage IV.

### Treatment delivery

There were 48 patients eligible and assessable for toxicity and response. All but one of these patients (98%) completed the concurrent treatment protocol that consisted of two cycles of chemotherapy and radiotherapy with a dose of 60 Gy. Because of grade 3 oesophagitis, one patient was unable to complete the scheduled concurrent chemoradiotherapy. In total, 43 (90%) patients received at least one cycle of consolidation chemotherapy. Consolidation chemotherapy was started on day 57 to day 81 (median, day 63), and 39 (81%) patients completed two cycles of consolidation chemotherapy.

A total of four patients were unable to complete the two cycles of consolidation chemotherapy because of toxicities (grade 3 pneumonitis, grade 5 cerebral haemorrhage, grade 3 fatigue, and delayed recovery of bone marrow function). In accordance with the study protocol, dose reductions were necessary in 8 out of 48 patients during concurrent treatment (17%) and in 14 out of 43 patients during consolidation chemotherapy (32%).

The projected dose intensities of cisplatin and S-1 were 15 and 280 mg m^−2^ per week, respectively. The actual dose intensity of cisplatin was 11.2–16 mg m^−2^ per week (mean: 13.8; 92% of the projected dose intensity), and the actual dose intensity of S-1 was 135–296 mg m^−2^ per week (mean, 243; 87% of the projected dose intensity).

The radiation dose intensity was 6.5–10 Gy per week (mean, 9.77; 97.7% of the projected dose intensity).

### Response to treatment

We observed that 24 out of 25 patients had a partial response in the first stage, and therefore an additional 25 patients were enrolled.

Of 48 patients, none had a complete response, whereas 42 patients had a partial response and 6 patients had stable disease. No patients had progressive disease. The overall response rate was 87.5% (95% CI: 79.1–96.9).

### Toxicity of treatment

Major grade 3 and 4 toxicities for the concurrent chemoradiotherapy phase are summarised in Table 2. Concurrent chemoradiotherapy was generally well tolerated. Although 5 out of the 48 (10%) assessable patients had grade 3 oesophagitis, none showed grade 4 radiation-associated oesophagitis. The most common grade 3/4 haematological toxicities were neutropenia (which included two patients with grade 3 febrile neutropenia) and leukopenia (25%). Grade 3/4 anaemia and thrombocytopenia were uncommon (two patients each, 4%) and no patients were observed to have grade 3/4 pneumonitis. Other grade 3/4 toxicities were anorexia (3 patients, 6%), fatigue (2 patients, 4%), diarrhoea (2 patients, 4%) and increases in serum alanine aminotransaminase or aspartate aminotransferase levels (one patient, 2%).

Table 2 also summarises the major grade 3 and 4 toxicities for the 43 patients who received consolidation chemotherapy. The most common grade 3/4 toxicity was neutropenia (which occurred in 4 (10%) patients, which included one patient with grade 3 febrile neutropenia). Grade 3 pneumonitis was observed in 2 (5%) patients. After steroid therapy, both patients responded and showed improvement.

One patient developed hemiparesis on day 34 of the first consolidation chemotherapy treatment. A brain CT scan showed multiple brain metastases with cerebral haemorrhage, and the patient died on the following day. Although no thrombocytopenia and/or neutropenia were observed, we could not exclude the possibility of treatment-related death.

### Survival

**Figure 1:**
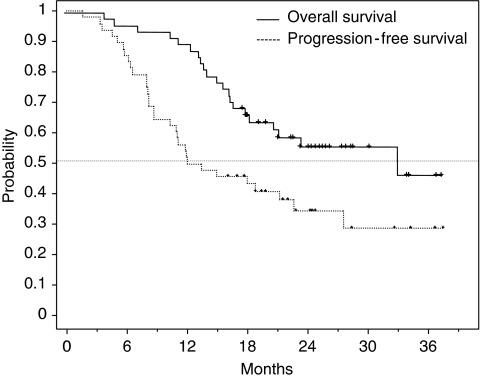
Kaplan–Meier overall survival (OS) and progression-free survival (PFS) curves in all patients. The median PFS was 12.0 months and the median OS was 33.1 months.

For the current analysis, the median follow-up time was 25.4 months (range, 12–37.6). Of 48 patients, 30 (62.5%) had recurrence of the disease, and to date, 21 of these patients have died. Figure 1 shows OS and PFS for the patients enrolled in the study. The median PFS was 12.0 months and the median OS was 33.1 months. The 1- and 2-year survival rates were 89.5 and 56%, respectively. No correlation was apparent between PFS or OS and sex, age, histology, disease stage, or smoking status.

**Figure 2:**
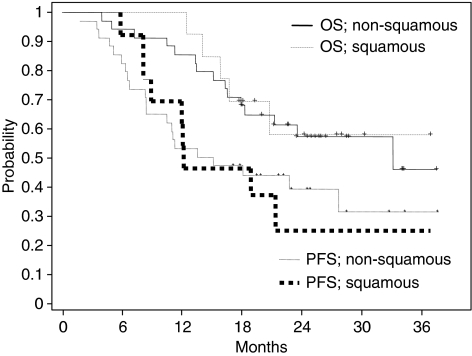
Kaplan–Meier overall survival (OS) and progression-free survival (PFS) curves for patients with non-squamous histology (adenocarcinoma plus large cell), and patients with squamous histology.

Figure 2 shows OS and PFS curves for the patients with squamous histology and those with non-squamous histology (adenocarcinoma plus other types). There were no differences noted in the survival curves between the squamous and non-squamous histologies.

Among 30 reported sites of first failure, 15 (50%) were distant, 12 (40%) were loco-regional, and 3 (10%) were both loco-regional and distant (Table 3). The brain and lung were the most common sites for distant metastases (17% each).

## Discussion

This is the first phase II study to investigate the use of the new oral fluoropyrimidine agent, S-1, in the curative-intent therapy setting of stage III NSCLC. Our data indicate positive results with only mild toxicities after treatment consisting of concomitant radiotherapy in addition to S-1 plus cisplatin administered at the same doses as those used for advanced metastatic NSCLC. The median OS of 33.1 months, and the 1- and 2-year survival rates of 89.5 and 56% are very encouraging.

The advantages of using concomitant chemoradiotherapy have been shown by both the West Japan Lung Cancer Group study trial, which evaluated mitomycin, vinblastine, and cisplatin regimens, and the similarly designed Radiation Therapy Oncology Group trial, which evaluated cisplatin and vinblastine (Furuse *et al*, 1999; Curran *et al*, 2003). The concurrent chemoradiotherapy arm in the Japanese study resulted in a median OS of 16.5 months with survival rates at 1 and 2 years of 64.1 and 34.6%, respectively (Furuse *et al*, 1999).

Recently, new agents, such as paclitaxel, vinorelbine, docetaxel, and gemcitabine, have been evaluated in several studies that administered the agents in combination with platinum when using an induction and/or concurrent strategy. However, for many of these newer agents, the dose-limiting toxicities require that lower doses be given during the concurrent phase (Choy *et al*, 1998; Vokes *et al*, 2002; Kiura *et al*, 2003; Belani *et al*, 2005; Fournel *et al*, 2005).

The median PFS and the median OS for these new concurrent regimens were 8.4–16.3 and 14.8–23.4 months, respectively. Overall survival rates at 1 and 2 years were 56.3–76 and 25–54%, respectively. These results are not satisfactory. Compared with these studies, the results of our study are exceptionally good.

The increased response rate and prolonged survival seen in this study could explain the radiation-sensitising effect resulting from prolonged exposure to S-1, because it was administered orally on a daily basis. The European Organization for Research and Treatment of Cancer reported a randomised trial that evaluated radiation alone, radiation plus cisplatin at 30 mg m^−2^, weekly, or radiation plus cisplatin at 6 mg m^−2^, daily (Schaake-Koning *et al*, 1992). Results showed an improvement in the OS for radiation plus daily cisplatin as against that for radiation alone. However, the analysis of the patterns of failure indicated there was improvement in regional control only. Therefore, prolonged exposure to S-1 may enhance the radiation effect and contribute to improved local control.

Alternatively, it could be argued that prolonged survival was due to substantial second-line treatment, especially epidermal growth factor receptor (EGFR) tyrosine kinase inhibitors (TKIs), because the median PFS in this study was 12.0 months, which is not exceptionally good compared with previous reports. There were 16 out of 30 relapsed patients receiving substantial second-line treatment. Although we did not analyse EGFR mutational status in this study, 7 out of 30 relapsed patients received EGFR-TKIs. Only one patient responded to an EGFR-TKI, hence the use of EGFR-TKIs cannot exclusively explain the prolonged survival, and it is unclear why we observed so much difference between the median PFS and OS.

Another advantage of using this regimen is that there are only mild toxicities and very few treatment interruptions. Overall, 98% of our study patients completed the concurrent chemoradiotherapy, and 90% of the patients received at least one cycle of consolidation chemotherapy, resulting in a total of 81% of patients completing the planned treatment.

Oesophagitis is the principal toxicity, but only 5 (10%) patients developed grade 3/4 oesophagitis. A possible reason for mild oesophagitis is that potassium oxonate may inhibit the formation of 5-FU nucleotides in the oesophagus, thereby reducing the radiation-sensitising effect in oesophagus rather than in the tumour.

In addition, we observed grade 2 and 3 pulmonary toxicity in only three and two patients, respectively, and there was no grade 5 pulmonary toxicity seen. The reason for the low incidence of severe radiation pneumonitis is unclear, and one possible explanation may be that it was due to the administration of TRT using three-dimensional treatment planning.

Recently, two reports on phase I studies of split-dose S-1 and cisplatin combined with concurrent radiotherapy were published (Kaira *et al*, 2008; Chikamori *et al*, 2009). However, this split-dose regimen using cisplatin and S-1 has not been investigated for advanced NSCLC, and we do not know the potential efficacy of this chemotherapy. In addition, we cannot consider our chemoradiotherapy regimen as full-dose chemoradiotherapy, because the actual dose intensities of cisplatin and S-1 in this study were less than the dose intensities of a previous phase II study (70 and 65% for cisplatin and S-1, respectively, compared with the doses in the study reported by Endo *et al*, 2006).

Differences in the toxicity profile of S-1 as a single agent were observed between Japanese and Caucasians in previous studies (van Groeningen *et al*, 2000; Hoff *et al*, 2003). Although haematological toxicities predominated in Japan, diarrhoea was the dose-limiting toxicity in Western countries. In our study, only Japanese patients were enrolled; the toxicity profile of this chemoradiotherapy regimen may be different in Caucasians.

There are several other new agents of interest for stage III NSCLC. In particular, pemetrexed, which is a potent inhibitor of thymidylate synthase, seems promising (Taylor *et al*, 1992; Schultz *et al*, 1999). A recent phase III study showed that pemetrexed plus cisplatin yielded efficacy results in advanced NSCLC comparable to those for gemcitabine plus cisplatin (Scagliotti *et al*, 2008). In addition, pemetrexed plus cisplatin has an excellent safety profile; a recent phase I study showed that a full dose of pemetrexed plus cisplatin in conjunction with full-dose concurrent TRT is well tolerated (Brade *et al*, 2008). In patients with squamous carcinoma, however, cisplatin plus pemetrexed resulted in significantly worse survival than cisplatin/gemcitabine (Scagliotti *et al*, 2008). Using pemetrexed in the stage III setting is disadvantageous because stage III NSCLC may include squamous carcinoma more frequently than is seen in stage IV NSCLC. Although S-1 is also a potent inhibitor of thymidylate synthase, we did not observe any differences in either the response rate or survival between the squamous and non-squamous groups. However, because of the small number of patients in this study, we are unable to draw any solid conclusions, and it will be necessary to conduct a larger study to compare the efficacies for the different histology types.

Although we observed a high response rate for this regimen, analysis of the initial sites of failure indicated that local failure still occurred in half of the patients with recurrences, and thus our results highlight the need for further improvement in local control. With regard to local control, currently an additional treatment of interest for stage III NSCLC is dose escalation of TRT that uses three-dimensional treatment planning. In a recent randomised phase II study by the Cancer and Leukemia Group B, this new strategy resulted in a median survival of 24 months and a 1-year survival rate of 66.7% (Socinski *et al*, 2008). Therefore, we are currently in the process of testing full-dose S-1 and cisplatin chemotherapy in conjunction with concurrent high-dose TRT that uses three-dimensional conformal radiation therapy.

In conclusion, the response rates and survival of the patients enrolled in this trial are most encouraging and compare favourably with the results from similar multimodality studies. A further randomised trial will be necessary to fully evaluate the usefulness of these current findings.

## Figures and Tables

**Table 1:** Patient characteristics

**Characteristics**	**No. of patients**
Total no. of patients	50
	
*Age, years*	
Median	63
Range	35–74
	
*Sex*	
Men	43
Women	7
	
*Performance status*	
0	36
1	14
	
*Histology*	
Adenocarcinoma	30
Squamous	14
Other	6
	
*Stage*	
IIIA	22
T1-3N2M0	22
IIIB	26
T1-3N3M0	9
T4N0-1M0	6
T4N2M0	9
T4N3M0	2
IV	2

**Table 2:** Major toxicities

	**Concurrent chemoradiotherapy (*N*=48)**				**Consolidation chemotherapy (*N*=43)**			
	**Grade 3**		**Grade 4**		**Grade 3**		**Grade 4**	
**Toxicity**	**No.**	**%**	**No.**	**%**	**No.**	**%**	**No.**	**%**
*Haematologic*								
Leukopenia	11	23	1	2	3	7	0	
Neutropenia	11	23	0		3	7	1	2
Febrile neutropenia	2	4	0		1	2	0	
Thrombocytopenia	1	2	1	2	0		0	
Anaemia	2	4	0		2	5	1	2
								
*Non-haematologic*								
Nausea	1	2	0		0		0	
Anorexia	3	6	0		2	5	0	
Fatigue	2	4	0		1	2	0	
ALT, AST	1	2	0		0		0	
Pneumonitis	0	0	0		2	5	0	
Oesophagitis	5	10	0		0		0	
Constipation	2	4	0		0		0	
Diarrhoea	2	4	0		0	0	0	
Cerebral haemorrhage							1 (Grade5)	2

ALT=alanine aminotransaminase; AST=aspartate aminotransferase; Concurrent=S-1 plus cisplatin, and TRT (*N*=48); Consolidation=S-1 plus cisplatin (*N*=43).

**Table 3:** Sites of initial failure

	**Patients**	
**First site of failure**	**No.**	**%**
Local	12	40
Local+distant	3	10
Distant	15	50
Brain	5	17
Lung	5	17
Liver	2	7
Bone	2	7
Adrenal	1	3
